# Predictability of indicators in local activation time mapping of ablation success for premature ventricular contractions

**DOI:** 10.1002/joa3.13148

**Published:** 2024-10-14

**Authors:** Takahiko Nagase, Takafumi Kikuchi, Shun Akai, Masafumi Himeno, Ryo Ooyama, Yoshinori Yoshida, Chiyo Yoshino, Takafumi Nishida, Takahisa Tanaka, Mitsunori Ishino, Ryuichi Kato, Masao Kuwada

**Affiliations:** ^1^ Department of Cardiology Higashiyamato Hospital Tokyo Japan

**Keywords:** bipolar prematurity, earliest isochronal map area, local activation time difference, premature ventricular contraction, unipolar prematurity

## Abstract

**Introduction:**

Differences in predictability of ablation success for premature ventricular contractions (PVCs) between earliest isochronal map area (EIA), local activation time (LAT) differences on unipolar and bipolar electrograms (⊿LAT_Bi‐Uni_), LAT prematurity on bipolar electrograms (LAT_Bi_), and unipolar morphology of QS or Q pattern remain unclear. We verified multiple statistical predictabilities of those indicators of ablation success on mapped cardiac surface.

**Methods:**

Thirty‐five patients with multiple PVCs underwent catheter ablation after LAT mapping using multipolar mapping catheters with unipolar‐based annotation. Patients were divided into success and failure groups based on ablation success on mapped cardiac surfaces. Discrimination ability, reclassification table, calibration plots, and decision curve analysis of 10 ms EIA (EIA_10ms_), ⊿LAT_Bi‐Uni_, and LAT_Bi_ were validated. Unipolar morphology was compared between success and failure groups.

**Results:**

Right ventricular outflow tract, aortic cusp, and left ventricle were mapped in 17, 10, and 8 patients, respectively. In 14/35 (40%) patients, successful ablation was performed on mapped cardiac surfaces. Area under the curve of receiver‐operating characteristic curve of EIA_10ms_, ⊿LAT_Bi‐Uni_, and LAT_Bi_ were 0.874, 0.801, and 0.650, respectively (EIA_10ms_ vs. LAT_Bi_, *p* =.014; ⊿LAT_Bi‐Uni_ vs. LAT_Bi_, *p* =.278; EIA_10ms_ vs. ⊿LAT_Bi‐Uni_, *p* =.464). EIA_10ms_ and ⊿LAT_Bi‐Uni_ demonstrated better predictability, calibration, and clinical utility on reclassification table, calibration plots, and decision curve analysis than LAT_Bi_. Unipolar morphology of QS or Q pattern did not correlate with ablation success (*p* =.518).

**Conclusion:**

EIA_10ms_ and ⊿LAT_Bi‐Uni_ more accurately predict ablation success for PVCs on mapped cardiac surfaces than LAT_Bi_ and unipolar morphology.

## Graphical abstract

Predictability of indicators of ablation success.

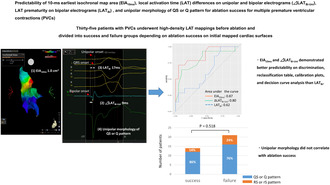

## 1 INTRODUCTION

Catheter ablation is established as an effective therapeutic choice for symptomatic multiple premature ventricular contractions (PVCs) or PVCs with left ventricular dysfunction.^1^, ^2^ Earliest isochronal map area (EIA), local activation time (LAT) differences on unipolar and bipolar electrograms (⊿LAT_Bi‐Uni_), LAT prematurity on bipolar electrograms (LAT_Bi_), and unipolar morphology of QS or Q pattern have been reported as indicators of ablation success.^3^, ^4^, ^5^, ^6^, ^7^, ^8^ Recently, high‐density mapping using multipolar mapping catheters has been shown to be effective for identifying origin of PVCs by accurate LAT mapping.^9^ Hower, those indicators have not been compared as prediction models, using multiple statistical analyses including discrimination, reclassification, calibration or clinical utility in the era of high‐density mapping.

Therefore, we verified the predictability of ablation success of EIA, ⊿LAT_Bi‐Uni_ and LAT_Bi_ obtained from high‐density LAT maps as prediction models along with correlation with ablation success and unipolar morphology of QS or Q pattern.

## 2 METHODS

### 2.1 Study subjects and design

From September 2018 to December 2023, 35 consecutive patients, in whom LAT maps of PVCs were made by multipolar mapping catheters in ablation of symptomatic multiple PVCs with CARTO3 system (Biosense Webster, Diamond Bar, CA), were enrolled. Cardiac surface estimated as the origin of PVCs based on the 12‐lead electrocardiogram was mapped depending on operators' discretion or previous reports.^10^, ^11^, ^12^, ^13^, ^14^ No patient underwent ablation based on the pace‐mapping. In this study, LAT maps of right ventricular outflow tract, left ventricle, and coronary cusps were initially created and evaluated. Regarding the mapped cardiac surfaces of left ventricle, left ventricular outflow tract, mitral valve annulus, and left ventricular posteroseptum were included.

The patients, in whom PVCs were abolished by ablation only on initial mapped cardiac surfaces, were defined as success group. Those, in whom ablation on the contralateral cardiac surfaces (e.g., left ventricular outflow tract or coronary cusps in the case of initial ablation surface of right ventricular outflow tract) was required or PVCs did not ultimately disappear, were classified into failure group. EIA, ⊿LAT_Bi‐Uni_, LAT_Bi_, and unipolar morphology of QS or Q pattern were compared offline after procedures between success and failure groups.

We assessed the following aspects of predictability of the indicators (1) discrimination ability, (2) reclassification, (3) calibration ability, and (4) clinical usefulness of 10 ms EIA (EIA10ms), ⊿LATBi‐Uni, and LATBi, and (5) differences on the rate of unipolar morphology of QS or Q pattern between success and failure groups. Regarding discrimination ability, area under the curve (AUC) by receiver‐operating characteristic curve was compared between the indicators and optimal cutoff value of each indicator was calculated. Net reclassification improvement (NRI) and integrated discrimination improvement (IDI) were evaluated on the reclassification tables. Calibration ability and clinical usefulness were validated on calibration plots and decision curve analysis, respectively. The study was approved by the local institutional review board (local ethics committee number: 2023–026) and was conducted according to the principles of the Declaration of Helsinki. All patients provided informed consent.

### 2.2 LAT mapping and ablation of PVCs

LAT maps of PVCs were created without isoproterenol infusion using DECANAV catheters (Biosense Webster) with 1 mm width electrodes and an interelectrode spacing of 2–8–2 mm for right ventricular outflow tract and 5‐spline PentaRay catheters (Biosense Webster) with 1 mm width electrodes and an interelectrode spacing of 2–6–2 mm or 8‐spline OctaRay catheters (Biosense Webster) with 0.5 mm width electrodes and an interelectrode spacing of 2–2–2‐2‐2 mm for left ventricles or coronary cusps. LAT maps were created with Pattern Matching Filter (Biosense Webster) using the algorithm annotating on the bipolar distal electrodes the timing of unipolar steepest dV/dT that coincides with a bipolar potential (WaveFront, Biosense Webster). Annotation reference was set based on 12‐lead electrocardiogram using the equipped algorithm (advanced reference annotation, Biosense Webster). Surface and intracardiac electrocardiograms were recorded using an EP‐system (RMC‐5000, Nihon Kohden, Tokyo, Japan).

After LAT mappings, earliest sites on bipolar electrograms were validated near earliest color areas using saline‐irrigated contact force sensing catheters (Thermocool SmartTouch Surround Flow, Biosense Webster). Ablation was initially performed on the mapped cardiac surfaces. Ablation sites were targeted at the bipolar earliest site with confirmation of unipolar morphology on the EP‐system. LAT maps were not converted to isochronal maps during the procedures. Ablation was performed with 25–40 W with a maximum temperature of 40°C within impedance drops of ≤15 ohm for 60–90 s. Ablation success site was defined as elimination of PVCs within 30 s. If ablation at the bipolar earliest site did not abolish PVCs, the adjacent earliest activation sites were cauterized. If 10–15 applications could not suppress PVCs, the bipolar earliest site of the adjacent contralateral cardiac surfaces were similarly ablated. No epicardial ablation except for that via intracoronary sinus was not performed. The procedural endpoint was defined as disappearance of PVCs after 30 min waiting time and administration of isoproterenol (1–3 μg/min).

### 2.3 Measurements of indicators

EIA_10ms_ was measured offline after LAT maps of PVCs were converted to isochronal maps with 10 ms steps. Obvious incorrect annotations were deleted. The minimum density of points required to conclude that the electroanatomic map of a given chamber was acceptable was defined as a fill threshold of 15 mm as previously described.^3^, ^4^

⊿LAT_Bi‐Uni_, LAT_Bi_, and unipolar morphology of QS or Q pattern were recorded offline at a sweep speed of 400 mm/s on CARTO3 system. The high‐ and low‐pass filters were 16 and 500 Hz, respectively, for bipolar electrograms and 2 and 240 Hz, respectively, for unipolar electrograms. Surface electrograms were filtered with a low‐pass of 0.5 Hz and a high‐pass of 120 Hz.

**FIGURE 1:**
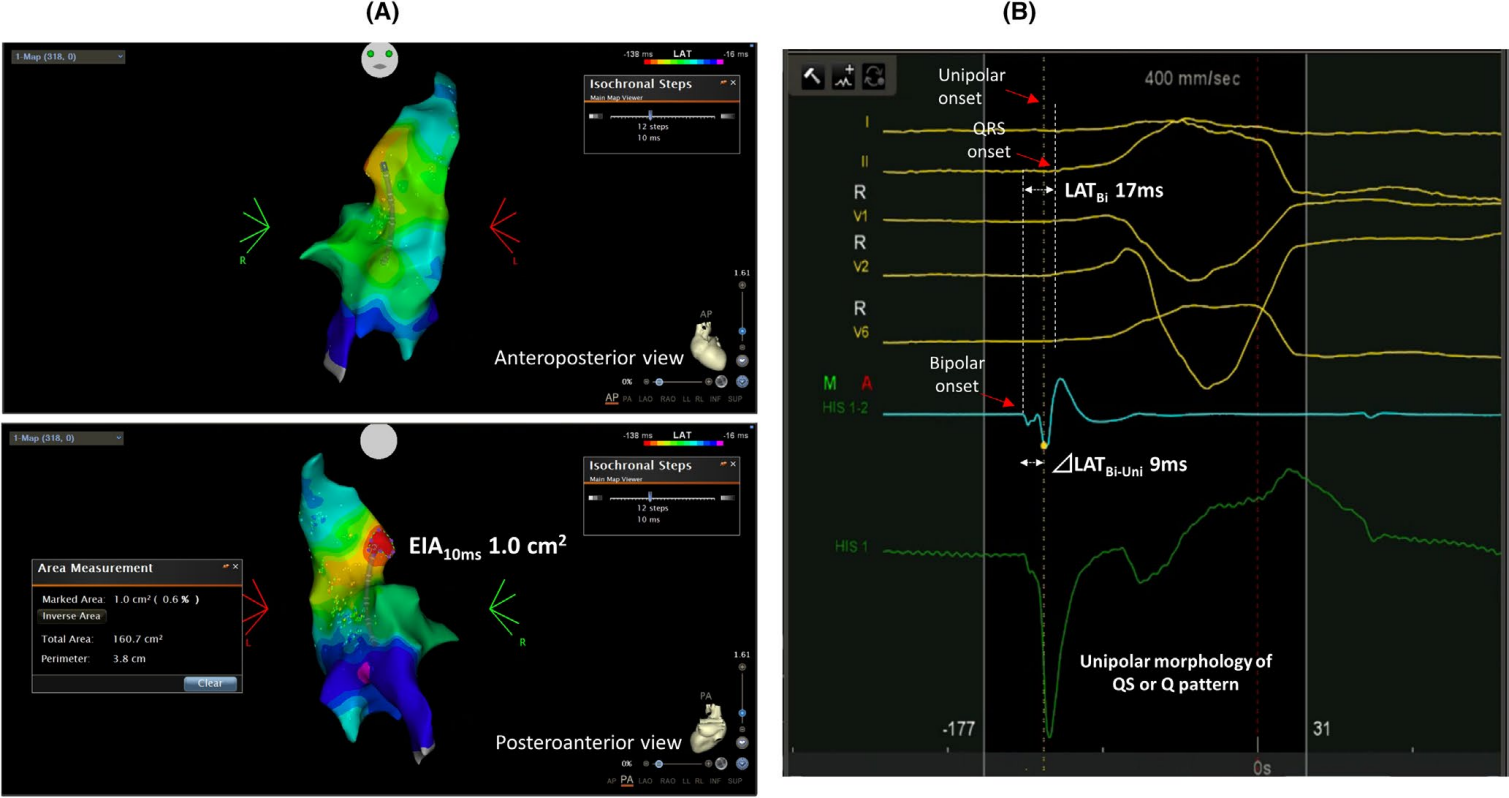
Measurements of indicators of ablation success. (A) An example of successful ablation on posterolateral wall of right ventricular outflow tract (upper, anteroposterior view; lower, posteroanterior view). (B) Intracardiac electrocardiograms at the point of earliest steepest dV/dT on unipole. ⊿LATBi‐Uni, local activation time (LAT) differences on unipolar and bipolar electrograms; EIA10ms, 10 ms earliest isochronal map area; LATBi, LAT prematurity on bipolar electrograms.

⊿LAT_Bi‐Uni_ was recorded at the point of earliest steepest dV/dT on unipole automatically annotated by WaveFront (Biosense Webster) and was defined as the absolute value of LAT difference between earliest LAT on bipole and timing of steepest dV/dT on unipole of the distal electrode of the same bipole. Because earliest sites by WaveFront (Biosense Webster) and manual bipolar annotation show good correlation,^15^ LAT_Bi_ was measured at or near the earliest sites identified by WaveFront (Biosense Webster) within EIA_10ms_ and was defined as LAT difference between earliest bipolar deflection and QRS onset. Unipolar morphology of QS or Q pattern was evaluated at the point of the earliest sites identified by WaveFront (Biosense Webster). All the indicators were assessed similarly both in both success and failure groups. An example of measurements is shown in Figure 1A,B. The correlation between EIA_10ms_, ⊿LAT_Bi‐Uni_, and LAT_Bi_ were also calculated.

### 2.4 Follow‐up

Antiarrhythmic drugs were discontinued after procedures. Patients attended outpatient visits 1 month after procedures and every 3 months thereafter. Twelve‐lead electrocardiograms at each visit and 24 h Holter monitoring every 6 months after procedures were evaluated for recurrence of PVCs.

### 2.5 Statistical analysis

Continuous variables with a normal distribution and nonparametric variables are presented as means ± standard deviation and the median and interquartile range, respectively. To compare continuous variables with normal distribution and nonparametric variables, we used Student *t* test and Mann–Whitney U test, respectively. Categorical variables are expressed as number and percentage and were compared using 𝜒^2^ test between success and failure groups.

Regarding sample size estimation, there is no generally accepted approach for the estimation of the sample size for derivation of risk prediction models.^16^ However, previous studies validating EIA or ⊿LAT_Bi‐Uni_ for ablation success analyzed 15–70 patients.^3^, ^4^, ^5^, ^6^ Also, Herczku et al.^3^ reported that the median differences and interquartile range of EIA_10ms_ on right ventricular outflow tract differentiating PVC origins of right or left ventricular outflow tracts was 2 cm^2^ and 1 cm^2^, respectively. Based on those values, the required sample size was calculated with detection difference of 2 cm^2^, standard deviation of 1 cm^2^, alpha error of 0.05, and power of 0.8, and was estimated as at least 34 patients.

Logistic regression models were prepared for prediction of ablation success on the mapped cardiac surfaces and were assessed by goodness‐of‐fit test using 𝜒^2^ test with *p* =.948, which indicated overall performance of predictabilities of evaluated indicators. Discrimination ability, cutoff values and reclassification of indicators of ablation success were validated as described above. Calibration ability was assessed by evaluating how much the slope of the calibration line (plotting the predicted probabilities vs. the observed probabilities) deviates from the ideal of 1.0 and intercept of 0.0. We used a bootstrapping procedure with 1000 samples drawn with replacement from the original sample to assess the internal validation of the model. Decision curve analysis to indicate the net benefit and clinical utility of the indicators were performed as previously described.^17^, ^18^ The correlation between EIA_10ms_, ⊿LAT_Bi‐Uni_, and LAT_Bi_ were calculated with Spearman's rank‐correlation coefficient. A *p* value of <.05 was considered statistically significant. All data were calculated using JMP version 16.2.0 (SAS Institute, Inc, Cary, NC) and R (version 4.2.0, The R Foundation for Statistical Computing).

## 3 RESULTS

### 3.1 Patient characteristics

In 40% of all patients, coronary artery disease or other structural heart disease are complicated. The ratio of those underlying diseases, left ventricular ejection fraction and brain natriuretic peptide did not differ between success and failure groups. Mean burden of PVCs on Holter electrocardiograms was 23% without any statistical differences between groups. Repeat procedures were involved in one patient in success group and in one patient in failure group.

Electrocardiographic features of PVCs were classified into three types; right bundle branch block plus inferior axis, left bundle branch block plus inferior axis, and right bundle branch block plus superior axis. The features of PVCs including R/S amplitude ratio, R/S duration ratio, and V2 S/V3 R amplitude ratio were similar between success and failure groups. Details are summarized in Table 1.

**TABLE 1:** Baseline characteristics.

	All patients *n* = 35	Success *n* = 14	Failure *n* = 21	*p* value*
Age (years)	61 ± 17	54 ± 18	65 ± 15	.087
Male	19 (54)	6 (43)	13 (62)	.268
Hypertension	16 (46)	5 (36)	11 (52)	.491
Diabetes mellitus	7 (20)	1 (7)	6 (29)	.203
Coronary artery disease	5 (14)	3 (21)	2 (10)	.369
Structural heart disease^a^	9 (26)	4 (29)	5 (24)	1.000
Atrial fibrillation	2 (6)	1 (7)	1 (5)	1.000
Left ventricular ejection fraction (%)	60 ± 8	62 ± 8	58 ± 6	.116
BNP (pg/ml)	97 (29–215)	90 (53–196)	25 (114–223)	.753
Previous ablation	2 (6)	1 (7)	1 (5)	1.000
PVC burden (%)	23.2 ± 10.7	22.2 ± 10.2	24.0 ± 11.2	.627
Electrogram characteristics of PVC				
RBBB pattern + inferior axis	14 (40)	4 (29)	10 (48)	.311
RBBB pattern + superior axis	2 (6)	1 (7)	1 (5)	1.000
LBBB pattern + inferior axis	19 (54)	9 (64)	10 (48)	.332
R/S amplitude ratio in V1	0.29 (0.13–1.67)	0.07 (0.16–2.25)	0.14 (0.67–3.25)	.162
R/S amplitude ratio in V2	0.15 (0.36–3.25)	0.30 (0.15–2.2)	0.71 (0.19–4.34)	.409
R/S duration ratio in V1	0.60 (0.50–2.00)	0.5 (0.38–1.25)	1 (0.50–2.00)	.057
R/S duration ratio in V2	1.00 (0.50–2.00)	0.50 (0.38–2.13)	1.00 (0.50–2.75)	.248
V2 S/V3 R amplitude ratio	2.00 (0.25–4.00)	1.8 (0.18–4.00)	3.15 (0.33–4.68)	.567

*Note*: Values are presented as mean ± standard deviation, n (%), or median (interquartile range).

Abbreviations: BNP, brain natriuretic peptide; LBBB, left bundle branch block; PVC, premature ventricular contraction; RBBB, right bundle branch block.

* Success vs. failure groups.

^a^ Structural heart diseases include valvular heart disease, postoperative state of ventricular septal defect, chronic heart failure with preserved ejection fraction, and dilated cardiomyopathy.

### 3.2 Procedural and mapping data and clinical outcome

PVCs were successfully ablated in 32/35 (91%) patients. Successful ablation sites were within EIA_10ms_ in all patients in success group. Ablation of contralateral cardiac surfaces was performed in all patients in failure group. Procedure and fluoroscopy time were longer in failure group than success group (both, *p* <.001). All patients were ablated through endocardial access. Ablation in coronary sinus was performed in only two patients in failure group.

Initial mapped cardiac surfaces were right ventricular outflow tract, left ventricle, and coronary cusps in 49%, 23%, and 29% of all patients, respectively, and did not differ between success and failure groups. Mapping points and fill thresholds of isochronal maps on the initial mapped cardiac surfaces were not different between the groups. EIA_10ms_ and ⊿LAT_Bi‐Un**i**_ were smaller in success group than in failure group (both, *p* <.01), while LAT_Bi_ was similar between groups. Details are summarized in Table 2.

**TABLE 2:** Procedural and mapping data.

	All patients *n* = 35	Success *n* = 14	Failure *n* = 21	*p* value*
Procedural data				
Successful PVC alation at the end	32 (91)	14 (100)	18 (86)	.259
Procedure time (min)	150 ± 39	129 ± 37	165 ± 34	.002
Fluoroscopy time (min)	20 ± 11	15 ± 9	28 ± 11	.002
Total complications	2 (6)	1 (7)	1 (5)	1.000
Access				
Endocardial	35 (100)	14 (100)	21 (100)	1.000
Epicardial	2 (6)	0 (0)	2 (10)	.506
Initial mapped cardiac surface				
Right ventricular outflow tract	17 (49)	8 (57)	9 (43)	.407
Left ventricular outflow tract/ Mitral valve annulus/posteroseptum	8 (23)	4 (29)	4 (19)	.685
Coronary cusps	10 (29)	2 (14)	8 (38)	.252
Mapping data				
Mapping points of initial mapped cardiac surface	262 (101–1024)	374 (99–1031)	222 (92–985)	.556
EIA_10ms_ (cm^2^)	2.1 (0.6–4.1)	0.5 (0.1–1.9)	3.7 (2.0–5.3)	.0002
⊿LAT_Bi‐Uni_ (ms)	13 (8–25)	8 (5–14)	20 (12–27)	.0030
LAT_Bi_ (ms)	13 (8–25)	15 (10–35)	12 (0–20)	.142
Fill threshold	7 (5–9)	8 (5–9)	7 (6–13)	.848

*Note*: Values are presented as *n* (%), mean ± standard deviation, or median (interquartile range).

Abbreviations: ⊿LAT_Bi‐Uni_, local activation time (LAT) differences on unipolar and bipolar electrograms; EIA_10ms_, 10‐ms earliest isochronal map area; IDI, integrated discrimination improvement; LAT_Bi_, LAT prematurity on bipolar electrograms; PVC, premature ventricular contraction.

* Success vs. failure groups.

During a mean follow‐up of 14 ± 8 months, PVCs were suppressed at PVC burden of <5% on 24‐h Holter monitoring in all patients with ablation success at the end of procedures.

### 3.3 Discrimination ability, reclassification table, and correlation of unipolar morphology with ablation success

**FIGURE 2:**
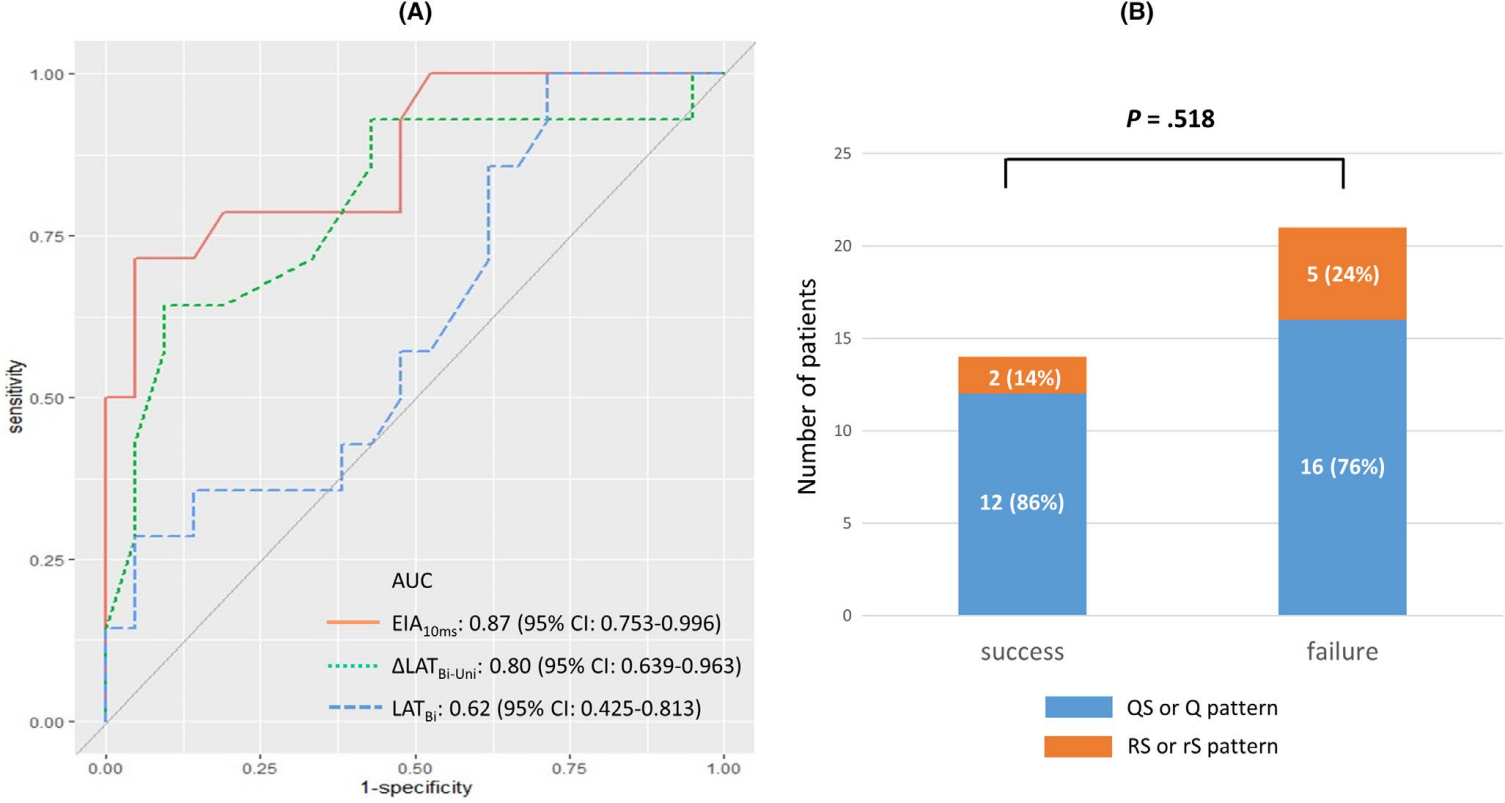
Receiver‐operating characteristic curve of indicators of ablation success (A) and unipolar morphology between success and failure groups (B). AUC, area under the curve; CI, confidence interval.

AUCs of EIA_10ms_, ⊿LAT_Bi‐Un**i**_, and LAT_Bi_, were 0.87, 0.80, and 0.62, respectively. Comparison of AUCs between each two indicators revealed the superiority of EIA_10ms_ on discrimination ability than LAT_Bi_ (*p* =.014; others, *p* = NS) (Figure 2A and Table 3). Receiver‐operating characteristic curve analysis demonstrated that optimal cutoff values for ablation success of EIA_10ms_, ⊿LAT_Bi‐Uni_, and LAT_Bi_ were 1.2 cm^2^, 9 ms, and 7 ms, respectively (Table 3). Regarding each cutoff values of EIA_10ms_ in different cardiac chambers, optimal cutoff values were 1.2 cm^2^, 1.5 cm^2^, and 0.1 cm^2^ in right ventricular outflow tract, left ventricle, and coronary cusps, respectively (right ventricular outflow tract, AUC 0.85, sensitivity 75.0%, specificity 88.9%; left ventricle, AUC 0.94, sensitivity 100.0%, specificity 75.0%; coronary cusps, AUC 0.69, sensitivity 50.0%, specificity 100.0%). However, the number of patients per cardiac chamber was small (especially for coronary cusps), and those analyses were only reference values.

**TABLE 3:** Discrimination ability and reclassification table of indicators of ablation success.

Comparison of AUC			*p* value
EIA_10ms_ vs. LAT_Bi_			.014
⊿LAT_Bi‐Uni_ vs. LAT_Bi_			.278
EIA_10ms_ vs. ⊿LAT_Bi‐Uni_			.464
Cutoff value of ablation success		Sensitivity (%)	Specificity (%)
EIA_10ms_ ≤1.2 cm^2^		71.4	95.2
⊿LAT_Bi‐Uni_ ≤9 ms		64.3	90.5
LAT_Bi_ ≥7 ms		100.0	33.4

Reclassification table			
(1) EIA_10ms_ vs. LAT_Bi_			
Outcome: absent	EIA_10ms_		
LAT_Bi_	[0, 0.5)	[0.5,1]	% reclassified
[0,0.5)	14	4	22
[0.5,1]	3	0	100
Outcome: present	EIA_10ms_		
LAT_Bi_	[0,0.5)	[0.5,1]	% reclassified
[0, 0.5)	3	7	70
[0.5, 1]	0	4	0
Combined data	EIA_10ms_		
LAT_Bi_	[0,0.5)	[0.5,1]	% reclassified
[0, 0.5)	17	11	39
[0.5, 1]	3	4	43
NRI (Continuous) (95% CI)	0.762 (0.140–1.384)		*p* Value: .016
IDI (95% CI)	0.294 (0.119–0.469)		*p* Value: .001
(2) ⊿LAT_Bi‐Uni_ vs. LAT_Bi_			
Outcome: absent	⊿LAT_Bi‐Uni_		
LAT_Bi_	[0,0.5)	[0.5,1]	% reclassified
[0, 0.5)	15	3	17
[0.5, 1]	2	1	67
Outcome: present	⊿LAT_Bi‐Uni_		
LAT_Bi_	[0,0.5)	[0.5,1]	% reclassified
[0, 0.5)	2	8	80
[0.5, 1]	3	1	75
Combined data	⊿LAT_Bi‐Uni_		
LAT_Bi_	[0,0.5)	[0.5,1]	% reclassified
[0, 0.5)	17	11	39
[0.5, 1]	5	2	71
NRI (Continuous) (95% CI)	0.714 (0.111–1.318)		*p* Value: .020
IDI (95% CI)	0.163 (−0.035–0.361)		*p* Value: .108
(3) EIA_10ms_ vs. ⊿LAT_Bi‐Uni_			
Outcome: absent	EIA_10ms_		
⊿LAT_Bi‐Uni_	[0,0.5)	[0.5,1]	% reclassified
[0, 0.5)	13	4	24
[0.5, 1]	4	0	100
Outcome: present	EIA_10ms_		
⊿LAT_Bi‐Uni_	[0,0.5)	[0.5,1]	% reclassified
[0, 0.5)	1	4	80
[0.5, 1]	2	7	22
Combined data	EIA_10ms_		
⊿LAT_Bi‐Uni_	[0,0.5)	[0.5,1]	% reclassified
[0, 0.5)	14	8	36
[0.5, 1]	6	7	46
NRI (Continuous) (95% CI)	0.810 (0.212–1.407)		*p* Value: .008
IDI (95% CI)	0.131 (−0.076–0.339)		*p* Value: .215

Abbreviations: AUC, area under the curve; CI, confidence interval; ⊿LAT_Bi‐Uni_, local activation time (LAT) differences on unipolar and bipolar electrograms; EIA_10ms_, 10‐ms earliest isochronal map area; IDI, integrated discrimination improvement; LAT_Bi_, LAT prematurity on bipolar electrograms; NRI, net reclassification improvement.

**FIGURE 3:**
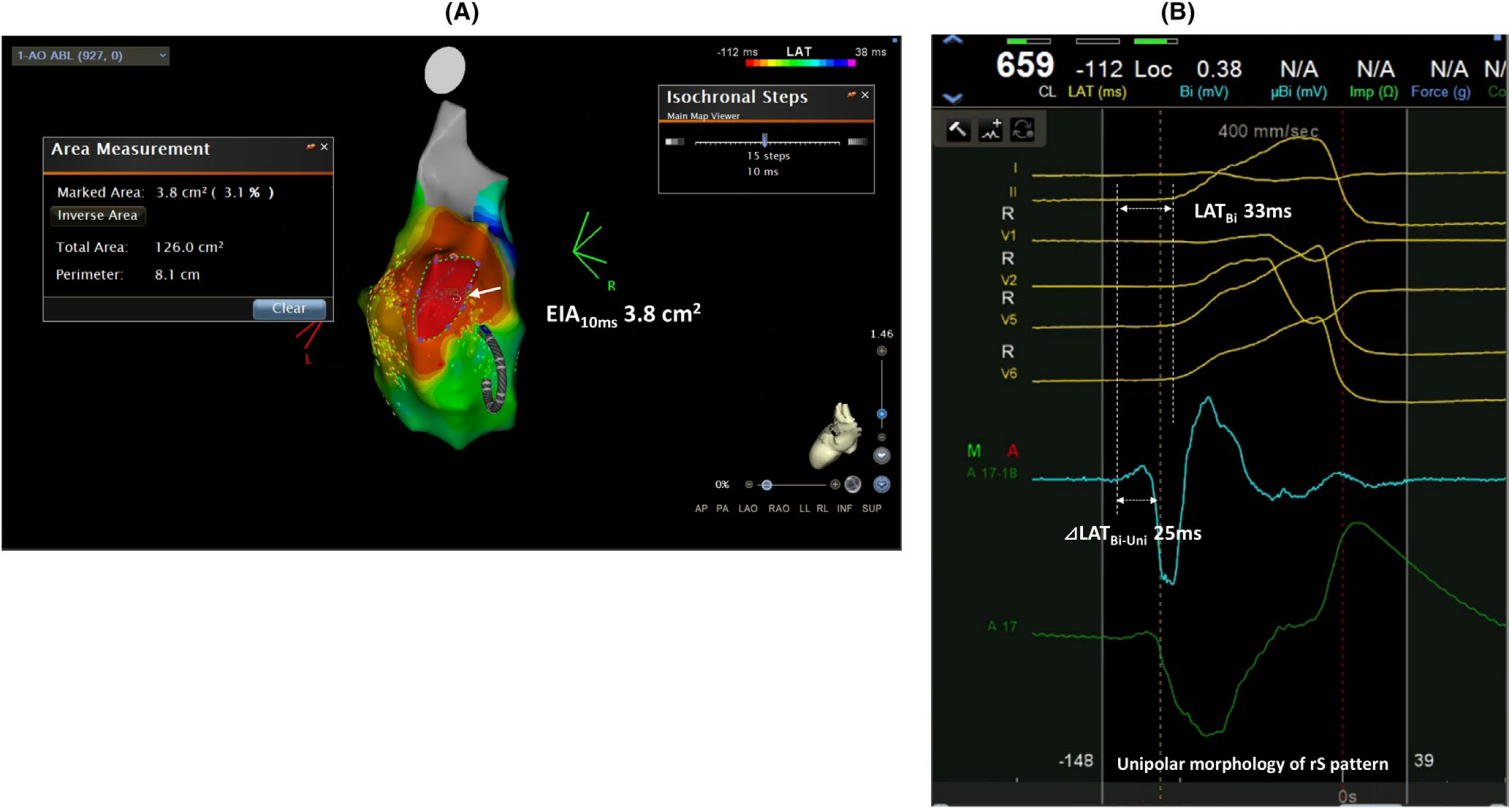
(A) An example of unsuccessful ablation on aortic cusps in failure group and the point of earliest steepest dV/dT on unipole (*white arrow*). (B) Intracardiac electrocardiograms at the point of earliest steepest dV/dT on unipole. ⊿LAT_Bi‐Uni_, Local activation time (LAT) differences on unipolar and bipolar electrograms; EIA_10ms_, 10 ms earliest isochronal map area; LAT_Bi_, LAT prematurity on bipolar electrograms.

In reclassification tables, EIA_10ms_ demonstrated better predictability for ablation success than ⊿LAT_Bi‐Uni_ and LAT_Bi_ for NRI and/or IDI (all, *p* <.05) (Table 3). ⊿LAT_Bi‐Uni_ showed better predictability on NRI than LAT_Bi_ (*p* =.020). Unipolar morphology did not correlate with ablation success (*p* =.518) (Figure 2B). An example of failure group is shown in Figure 3.

### 3.4 Calibration plots and decision curve analysis

**FIGURE 4:**
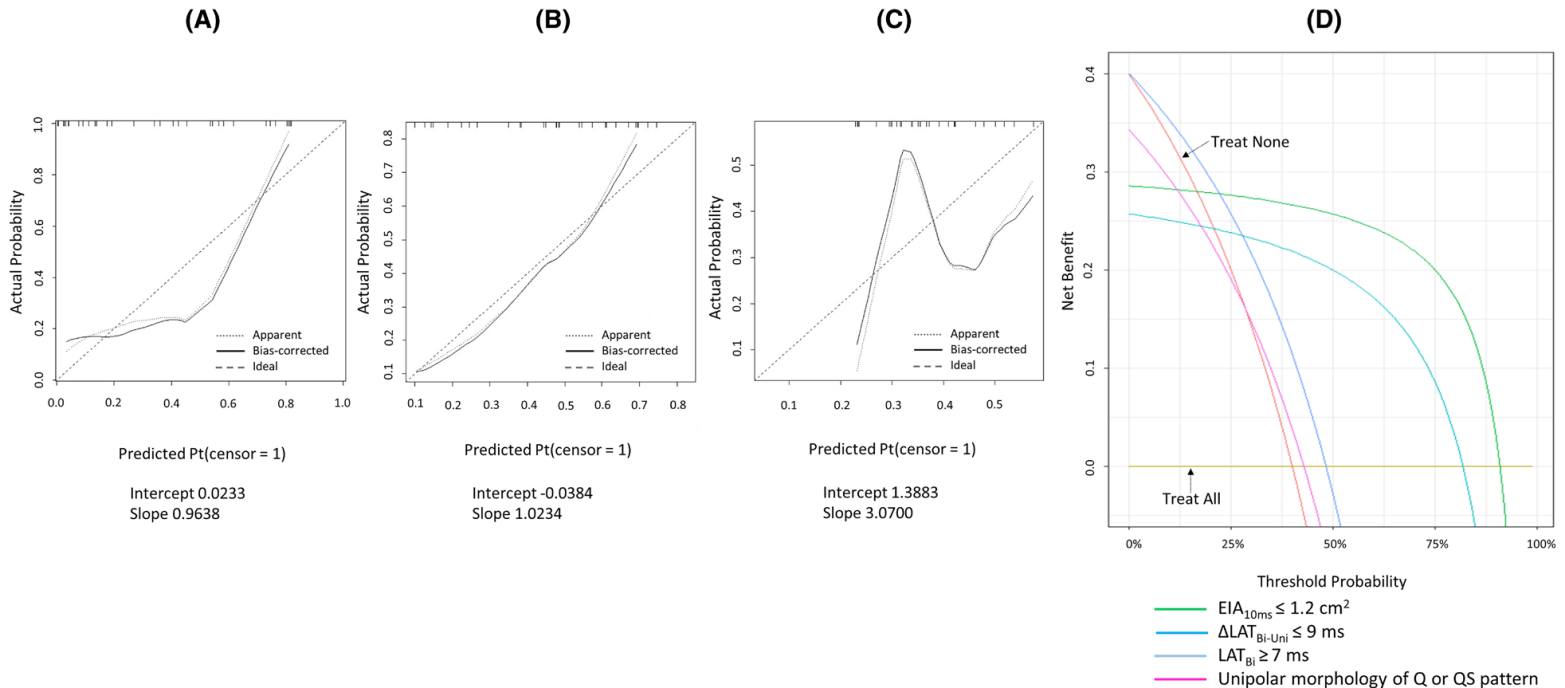
Calibration plots of 10 ms earliest isochronal map area (EIA_10ms_) (A), local activation time (LAT) differences on unipolar and bipolar electrograms (⊿LAT_Bi‐Uni_) (B), and LAT prematurity on bipolar electrograms (LAT_Bi_) (C). (D) Decision curve analysis of indicators for ablation success.

Calibration plot of each indicator is shown in Figure 4A–C. Along with better calibration slope and intercept, EIA_10ms_ and ⊿LAT_Bi‐Uni_ visually represented better correlation with the predicted probabilities vs. the observed probabilities compared with LAT_Bi_.

Decision curve analysis of the indicators including unipolar morphology revealed that net benefit was higher in order of small EIA_10ms_, small ⊿LAT_Bi‐Uni_, LAT_Bi_, and unipolar morphology of QS or Q pattern (Figure 4D). small EIA_10ms_, small ⊿LAT_Bi‐Uni_, LAT_Bi_ were defined by cutoff values from receiver‐operating characteristic curve analyses.

### 3.5 Correlation of indicators

**FIGURE 5:**
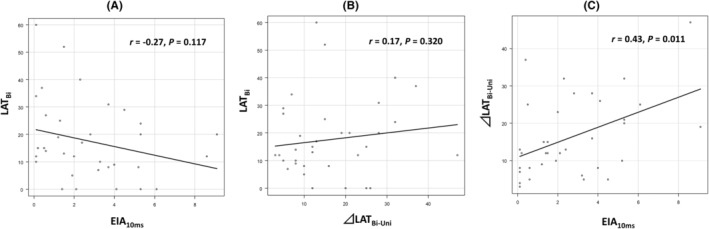
Correlation of 10 ms earliest isochronal map area (EIA_10ms_) and local activation time (LAT) prematurity on bipolar electrograms (LAT_Bi_) (A), LAT differences on unipolar and bipolar electrograms (⊿LAT_Bi‐Uni_) and LAT_Bi_ (B), and EIA_10ms_ and ⊿LAT_Bi‐Uni_ (C).

The correlation calculated with Spearman's rank‐correlation coefficient revealed mild correlation with EIA_10ms_ and LAT_Bi_ and with EIA_10ms_ and ⊿LAT_Bi‐Uni_. However, EIA_10ms_ and ⊿LAT_Bi‐Uni_ demonstrated almost no correlation (Figure 5A–C).

## 4 DISCUSSION

### 4.1 Major findings

This study validated indicators of ablation success for PVCs on high‐density mapped cardiac surfaces as prediction models. Main findings are as follows: (1) EIA_10ms_ showed better discrimination ability on receiver‐operating characteristic curve analysis than LAT_Bi_; (2) optimal cutoff values for ablation success of EIA_10ms_, ⊿LAT_Bi‐Uni_, and LAT_Bi_ by receiver‐operating characteristic curve analysis were 1.2 cm^2^, 9 ms, and 7 ms, respectively; (3) reclassification table, calibration plots, and decision curve analysis demonstrated better predictability of EIA_10ms_ and ⊿LAT_Bi‐Uni_; and (4) unipolar morphology of QS or Q pattern did not necessarily guarantee ablation success. To our knowledge, this study is the first to compare the predictability of EIA and the other indicators of ablation success for PVCs.

### 4.2 Previous reports

Herczku et al.^3^ reported that EIA_10ms_ of ≤2.3 cm^2^ on right ventricular outflow tract predicts ablation success on right ventricular outflow tract in 15 patients. Suzuki et al.^4^ showed that 5‐ms EIA of ≤0.7 cm^2^ predicts ablation success for PVC in 29 patients. LAT was recorded with a 3.5‐mm irrigation catheter tip (NaviStar, Biosense Webster; Thermocool SF, Biosense Webster) in those studies, leading to limited median mapping points of 40 to 99.^3^, ^4^ On the other hand, high‐density LAT mapping was performed using multielectrode catheters in this study. Therefore, optimal cut‐off value of EIA_10ms_ for ablation success was 1.2 cm^2^ (≤2.3 cm^2^), which reflects more accurate LAT maps.

The other reports stressed note in interpretation of unipolar signals for ablation success for PVCs.^5^, ^6^ Sabzwari et al.^5^ demonstrated that ⊿LAT_Bi‐Uni_ ≥ 15 ms in LAT map on right ventricular outflow tract predicts origin of left ventricular outflow tract with AUC of 0.77. Higuchi et al.^6^ described that the first rapid bipolar deflection that corresponds to a similarly early unipolar deflection was important for ablation success. Those reports demonstrated the importance of characteristics of unipolar potential (i.e., prematurity or sharpness) rather than type of unipolar morphology (i.e., QS or Q pattern). However, these indicators have not been compared as prediction models. Therefore, actual usefulness remained unclear.

### 4.3 Importance of analysis of indicators as prediction models

Evaluation methods of prediction models include overall performance, discrimination, reclassification, calibration, and clinical usefulness.^19^ Receiver‐operating characteristic curve analysis is one of the aspects and cannot necessarily reflect clinical utility.^20^ Therefore, NRI and IDI have been complimentarily validated for prediction models.^21^ However, NRI and IDI also have limitation for evaluation of predictability.^22^ Hilden et al.^22^ described that poorly calibrated models may appear advantageous, if NRI and IDI are used to measure gain in prediction performance. Hence, calibration plots and decision curve analysis were also evaluated. Calibration means how accurately prediction models correlate with clinical outcome.^19^, ^23^ Decision curve analysis is reported as the method to validate clinical usefulness of prediction models using net benefit.^18^

### 4.4 Clinical implications

Several indicators on 12‐lead electrocardiograms for diagnosis of origins of PVCs have been reported.^10^, ^11^, ^12^, ^13^, ^14^ However, those indicators can be influenced by anatomical varieties or structural heart diseases. On the other hand, recent innovations in multipolar mapping catheters provide rapid and more accurate LAT maps.^9^ The automated algorithm annotating unipolar steepest dV/dT equipped in CARTO3 system also can visualize stable LAT maps. Furthermore, evaluation of EIA_10ms_, ⊿LAT_Bi‐Uni_, and LAT_Bi_ in high‐density LAT maps could enable avoiding unnecessary ablation on mapped cardiac surfaces, leading to safer and shorter procedures.

### 4.5 Study limitations

First, the number of patients was relatively small, although sample size was estimated. Second, internal and external validation were not necessarily performed for all statistical methods as previously described,^24^ leading to possibility of statistical overfitting of evaluated data. Third, prematurity of LAT_Bi_ in mapped cardiac surfaces and the contralateral cardiac surfaces was not simultaneously compared for detecting optimal ablation site as previously reported.^25^ Fourth, EIA could be affected by the burden of PVCs. Finally, local variations of cardiac surfaces regarding running of cardiac fibers, diseased areas with structural heart diseases, or epicardial fat can influence on varieties of the indicators. In PVCs originating from left ventricular summit, eccentric activation patterns were reported in some cases,^26^ which might affect EIA.

## 5 CONCLUSION

EIA_10ms_ and ⊿LAT_Bi‐Uni_ on mapped cardiac surfaces demonstrated better predictability of ablation success for PVC than LAT_Bi_ and unipolar morphology of QS or Q pattern.

## FUNDING INFORMATION

This research did not receive any specific grant from funding agencies in the public, commercial, or not‐for‐profit sectors.

## CONFLICT OF INTEREST STATEMENT

All the authors declare no conflicts of interest.

## ETHICS STATEMENT

The study protocol complied with the Declaration of Helsinki and was approved by the Institutional Review Board of the Higashiyamato Hospital (local ethics committee number: 2023–026).

## CONSENT

All patients provided written informed consent.

## Data Availability

The data underlying this article will be shared on reasonable request to the corresponding authors.
